# Recurrent evolution of gut symbiotic bacteria in pentatomid stinkbugs

**DOI:** 10.1186/s40851-016-0061-4

**Published:** 2016-11-30

**Authors:** Takahiro Hosokawa, Yu Matsuura, Yoshitomo Kikuchi, Takema Fukatsu

**Affiliations:** 1 National Institute of Advanced Industrial Science and Technology (AIST), Tsukuba, 305-8566 Japan; 2 Faculty of Science, Kyushu University, Fukuoka, 819-0395 Japan; 3 Tropical Biosphere Research Center, University of the Ryukyus, Okinawa, 903-0213 Japan; 4 National Institute of Advanced Industrial Science and Technology (AIST), Hokkaido Center, Sapporo, 062-8517 Japan; 5 Department of Biological Sciences, Graduate School of Science, University of Tokyo, Tokyo, 113-0033 Japan; 6 Graduate School of Life and Environmental Sciences, University of Tsukuba, Tsukuba, 305-8572 Japan

**Keywords:** Stinkbug, Pentatomidae, Gut symbiont, γ-Proteobacteria, 16S rRNA gene, Molecular evolution, Reductive genome evolution

## Abstract

**Background:**

Diverse animals are intimately associated with microbial symbionts. How such host–symbiont associations have evolved is a fundamental biological issue. Recent studies have revealed a variety of evolutionary relationships, such as obligatory, facultative, and free-living, of gut bacterial symbiosis within the stinkbug family Pentatomidae, although the whole evolutionary picture remains elusive.

**Results:**

Here we investigated a comprehensive assembly of Japanese pentatomid stinkbugs representing 28 genera, 35 species, and 143 populations. Polymerase chain reaction (PCR), cloning, and sequencing of bacterial 16S rRNA gene from their midgut symbiotic organ consistently detected a single bacterial species from each of the insect samples, indicating a general tendency toward monosymbiotic gut association. Bacterial sequences detected from different populations of the same species were completely or nearly identical, indicating that the majority of the gut symbiotic associations are stably maintained at the species level. Furthermore, bacterial sequences detected from different species in the same genus tended to form well-supported clades, suggesting that host–symbiont associations are often stable even at the genus level. Meanwhile, when we compared such sequences with published sequences available in DNA databases, we found a number of counter-examples to such stable host–symbiont relationships; i.e., symbionts from different host species in the same genus may be phylogenetically distant, and symbionts from the same host species may be phylogenetically diverse. Likewise, symbionts of diverse pentatomid species may be closely related to symbionts of other stinkbug families, and symbionts of diverse pentatomid species may even be allied to free-living bacteria. Molecular evolutionary analyses revealed that higher molecular evolutionary rates, higher AT nucleotide compositions, and smaller genome sizes tended to be associated with the pentatomid symbionts constituting the stable lineages, whereas these traits were rarely observed in the pentatomid symbionts of promiscuous type.

**Conclusions:**

These results indicate that gut symbiotic bacteria have evolved repeatedly and dynamically in the stinkbug family Pentatomidae, which have plausibly entailed frequent symbiont acquisitions, losses, replacements and transfers, while establishing a number of relatively stable host-symbiont associations. The diverse host-symbiont relationships observed in the Pentatomidae will provide an ideal arena for investigating the evolution of symbiosis experimentally and theoretically.

**Electronic supplementary material:**

The online version of this article (doi:10.1186/s40851-016-0061-4) contains supplementary material, which is available to authorized users.

## Background

Many insects that rely on nutritionally limited dietary resources, such as plant sap, vertebrate blood, and woody material, are obligatorily associated with beneficial symbiotic microorganisms harbored within the gut lumen, body cavity, or cells. Without these microbial associates, the host insects are often unable to grow, survive, or reproduce normally. Conversely, symbiotic microorganisms are often unable to proliferate outside the host body, presumably because of adaptation to the intra-host environment. In this way, the host insect and the microbe are integrated into an almost inseparable biological entity, constituting an intimate symbiotic system of obligate nature [1–3]. Originally, however, such highly specialized symbiotic bacteria must have been derived from less-specialized free-living bacteria. The origins and mechanisms underlying the obligate host-symbiont associations are of fundamental ecological and evolutionary interest.

The majority of plant-sucking stinkbugs (Insecta: Hemiptera: Pentatomoidea) possess a symbiotic organ in a posterior region of the midgut, in which numerous crypts develop and harbor a dense population of specific symbiotic bacteria [1, 4–7]. In these stinkbugs, reproducing females excrete symbiont-containing materials from the anus to the eggs or newborns, which are exploited by the offspring to establish vertical symbiont transmission. When experimentally deprived of symbiotic bacteria, host stinkbugs suffer substantial fitness defects, including retarded growth, elevated mortality, morphological abnormalities, reduced offspring, and/or complete sterility, indicating the biological importance of this symbiosis [1, 8–24].

Despite the general importance of symbiotic bacteria for host stinkbugs, evolutionary patterns of the host–symbiont relationships differ markedly in different stinkbug lineages. In the families Plataspidae, Acanthosomatidae and Urostylididae, for example, the symbiont phylogeny mirrors the host phylogeny and the symbiont genomes are drastically reduced to less than 1 Mb, indicating stable host-symbiont co-speciation and degenerative genome evolution based on strict vertical symbiont transmission over evolutionary time [11, 12, 18]. In the families Pentatomidae and Cydnidae, by contrast, the symbiont phylogeny is not concordant with the host phylogeny, and the symbiont genomes exhibit no or moderate size reduction, suggesting relatively younger host–symbiont associations through acquisitions, horizontal transfers, and/or replacements of the symbiotic bacteria [13, 17, 20, 22, 23, 25]. Recent studies have revealed the striking diversity and dynamic evolutionary trajectories of gut symbiotic bacteria in the stinkbug family Pentatomidae: all gut symbiotic bacteria belong to the Enterobacteriaceae of the γ-Proteobacteria [10, 13–15, 20–24, 26–28]; however, the symbiotic bacteria are polyphyletic and their phylogeny does not reflect the phylogeny of their host stinkbugs, suggesting multiple evolutionary origins of the symbiotic bacteria from *Pantoea* spp. and allied γ-proteobacteria [13, 20, 22, 23, 27, 28]; some symbionts are uncultivable whereas others can be cultivated outside the host body [22]. In some cases, multiple obligate symbiotic bacteria may coexist in the same host species, different stinkbug species may share the same symbiont species, and some environmental bacteria can establish stable infection and mutualistic association with these stinkbug species [22]. A more comprehensive picture of the evolutionary dynamics of the symbiotic bacteria in the Pentatomidae is thus desirable.

In this study, we surveyed the gut symbiotic bacteria of a comprehensive assemblage of pentatomid stinkbugs representing 28 genera, 35 species, and 143 populations in Japan, by which their diversity, phylogenetic relationship, and molecular and genomic evolution were investigated.

## Materials

### Insect samples

The stinkbug samples examined in this study are listed in Additional file 1. These insects were collected in Japan mostly as adults, and either taken to the laboratory alive or preserved in acetone [29].

### DNA analyses

Fresh insects were individually dissected in phosphate buffered saline (154 mM NaCl, 1.06 mM KH_2_PO_4_, 2.97 mM Na_2_HPO_4_) with fine forceps under a dissection microscope. Acetone-preserved insects were dissected in the same way in 70% ethanol. Each dissected symbiotic organ of the midgut fourth section with numerous symbiont-harboring crypts was subjected to DNA extraction using QIAamp DNA Mini kit (Qiagen). A 1.5 kb segment of bacterial 16S rRNA gene was amplified by PCR using primers 16SA1 (5′-AGA GTT TGA TCM TGG CTC AG-3′) and 16SB1 (5′-TAC GGY TAC CTT GTT ACG ACT T-3′), and cloned and sequenced as described [30]. The nucleotide sequences determined in this study were deposited in the DNA Data Bank of Japan (http://www.ddbj.nig.ac.jp/index-e.html) under accession numbers LC168475-LC168617 (see Additional file 1).

### Molecular phylogenetic and evolutionary analyses

Multiple alignments of the nucleotide sequences were constructed by the program MAFFT v7.271 (G-INS-i) [31] and gap-containing regions and ambiguous sites of the alignment were removed manually. The nucleotide substitution model, GTR + I + G, was selected using the program jModeltest 2 based on the Akaike information criterion [32, 33]. The phylogenetic analyses were conducted by Bayesian and maximum-likelihood (ML) methods using the programs MrBayes v3.2.6 [34] and RAxML v8.1.5 [35], respectively. In the Bayesian analysis, multiple independent runs with four simultaneous Markov chains were performed for 50,000,000 generations, producing 50,001 trees (sample freq = 1,000) in each run. After discarding the first 12,501 samples as ‘burn-in’, a total of 75,000 trees were used to generate majority rule consensus trees and calculate the posterior probabilities. In the ML analysis, the best-scoring ML tree was searched and the supported values were calculated by 1000 rapid bootstraps. Calculations of genetic distances and relative rate tests were performed by the program RRTree [36].

## Results and discussion

### 16S rRNA gene sequences of gut symbiotic bacteria of pentatomid stinkbugs

From the midgut symbiotic organs of all 143 stinkbug individuals, representing 28 genera and 35 species, bacterial 16S rRNA gene was subjected to PCR amplification, cloning and sequencing. For each sample, five clones were sequenced and all yielded the same sequence, indicating monosymbiotic status of pentatomid stinkbugs in general. Within the same species, the sequences were either completely identical (most of the species) or nearly identical (ex. *Alcimocoris japonensis* > 99.86%; *Dybowskyia reticulate* > 99.86%; *Glaucias subpunctatus* > 99.86%; *Gonopsis affinis* > 99.93%; *Graphosoma rubrolineatum* > 99.93%) (Additional file 1). Within the same genus, sequences from different species tended to show high similarities to each other (ex. *Homalogonia* spp. 98.64%; *Menida* spp. 93.89–98.10%; *Pentatoma* spp. 98.29%; *Scotinophara* spp. 97.69–98.23%).

### Molecular phylogenetic analysis of gut symbiotic bacteria of pentatomid stinkbugs

**Fig. 1:**
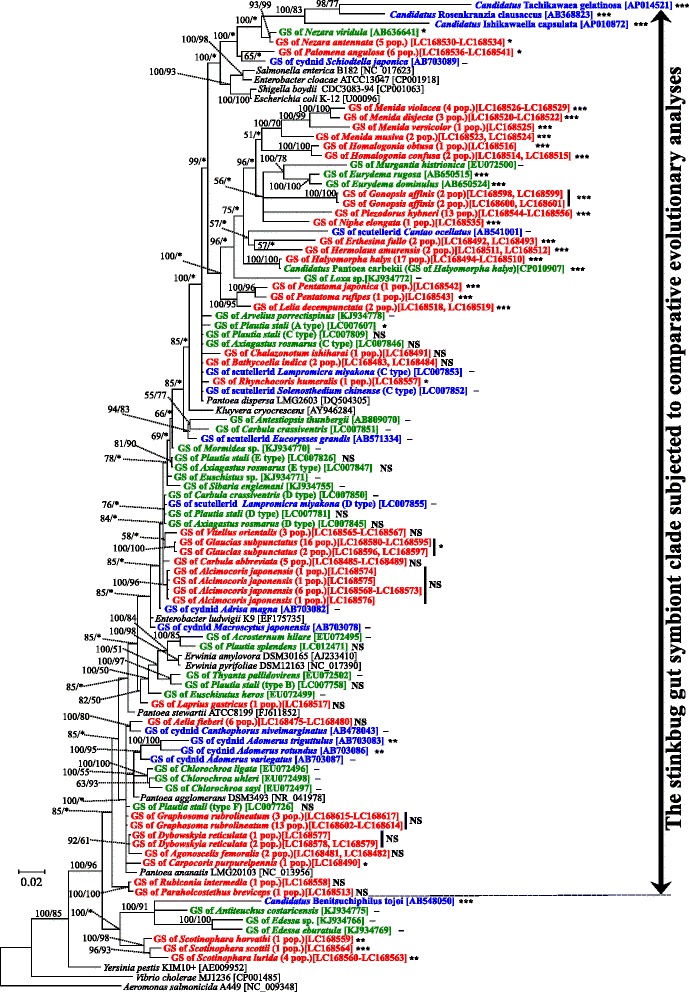
Phylogenetic relationship of gut symbiotic bacteria from stinkbugs of the family Pentatomidae, gut symbiotic bacteria from other stinkbug groups, and allied free-living bacteria of the Enterobacteriaceae in the γ-Proteobacteria. A Bayesian phylogeny inferred from 1260 aligned nucleotide sites of 16S rRNA genes is shown with statistical support values for each node (posterior probability of Bayesian analysis/bootstrap probability of maximum-likelihood analysis; asterisks indicate values lower than 50%). Colors indicate the following bacterial categories: *red*, gut symbiotic bacteria of pentatomid stinkbugs determined in this study; *green*, gut symbiotic bacteria of pentatomid stinkbugs determined in previous studies; *blue*, gut symbiotic bacteria reported from other stinkbug families; *black*, free-living γ -proteobacteria. “GS” and “pop.” indicate gut symbiont(s) and population(s), respectively; accession numbers are shown in brackets. For example, “GS of *Nezara antennata* (5 pop.) [LC168530-LC168534]” means “16S rRNA gene sequences of gut symbiotic bacteria of *Nezara antennata* representing five populations with sequence accession numbers LC168530-LC168534”. Statistical significance levels of the relative rate tests (see Additional file 3 and Additional file 4) are labeled on the right side of each stinkbug gut symbiont sequence as: ***, *P* < 0.001; **, *P* < 0.01; *, *P* < 0.05; NS, *P* > 0.05; −, not analyzed. “The stinkbug gut symbiont clade subjected to comparative evolutionary analyses” is shown on the right side of the phylogeny

In addition to the 16S rRNA gene sequences of the gut symbiotic bacteria of the pentatomid stinkbugs determined in this study, we retrieved already-published 16S rRNA gene sequences of gut symbiotic bacteria of pentatomid stinkbugs [13–15, 20–22, 26, 28] (Additional file 2), those of gut symbiotic bacteria of other stinkbugs representing the families Scutelleridae, Cydnidae, Parastrachiidae, Acanthosomatidae, Plataspidae and Urostylididae [15, 18, 25, 37–41] (Additional file 2), and those of closely-related free-living bacteria [42–52] (Additional file 2). Molecular phylogenetic analysis revealed that the gut symbionts of the pentatomid stinkbugs were all placed within the Enterobacteriaceae of the γ-Proteobacteria (Fig. 1). Of these, the gut symbionts of *Scotinophara* spp. constituted a distinct basal clade, clustering with the gut symbionts of *Edessa* spp. and the genome-reduced gut symbiont, *Candidatus* Benitsuchiphilus tojoi, of the stinkbug family Parastrachiidae. The other stinkbug gut symbionts formed a large and coherent cluster together with such free-living γ-proteobacteria as *Pantoea*, *Enterobacter*, *Erwinia*, etc. Among them, the gut symbionts of *Nezara* spp. clustered with the genome-reduced gut symbionts of other stinkbug families, *Candidatus* Ishikawaella capsulata of the Plataspidae, *Candidatus* Rosenkranzia clausaccus of the Acanthosomatidae, and *Candidatus* Tachikawaea gelatinosa of the Urostylididae. Outside of this cluster were placed the gut symbiont of *Palomena angulosa* and the gut symbiont of a cydnid stinkbug, and further outside of them were placed *Escherichia coli* and allied free-living γ-proteobacteria (Fig. 1).

### Intraspecific uniformity and exceptional diversity of gut symbiotic bacteria among pentatomid stinkbugs

As described above, bacterial 16S rRNA gene sequences obtained from different individuals of the same species were completely or nearly identical across different populations for most of the pentatomid species examined (Fig. 1). It should be noted, however, that several exceptional cases were observed in *Plautia stali*, *Axiagastus rosmarus* and *Carbula crassiventris*, as reported in a previous study [22], wherein different individuals and populations of the same pentatomid species may be associated with distinct bacterial symbionts (Fig. 1).

### Intrageneric coherence and diversity of gut symbiotic bacteria among pentatomid stinkbugs

In the phylogeny, the gut symbiotic bacteria of different pentatomid species belonging to the same genus tended to be closely related to each other (ex. *Homalogonia* spp., *Menida* spp., *Nezara* spp., *Pentatoma* spp., *Scotinophara* spp., etc.) (Fig. 1), as had been recognized in previous studies (ex. *Chlorochroa* spp., *Eurydema* spp., *Edessa* spp., etc.) [13, 15, 20]. On the other hand, some pentatomid species belonging to the same genus were associated with phylogenetically distinct gut symbiotic bacteria (ex. *Carbula abbreviata* vs. *C. crassiventris*, *Euschistus heros* vs. *E.* sp., *Plautia stali* vs. *P. splendens*, etc.) [13, 20–22] (Fig. 1).

### Multiple evolutionary origins of gut symbiotic bacteria among pentatomid stinkbugs

These phylogenetic patterns indicate that the gut symbiotic bacteria are polyphyletic in the stinkbug family Pentatomidae, as has been suggested in previous studies [13, 20, 22, 23]. Among diverse pentatomid species, presumably, their gut symbiotic bacteria have evolved in a dynamic manner, repeatedly acquired from environmental bacteria, horizontally transferred from different stinkbug species, and/or replacing pre-existing symbiotic bacteria. For further understanding of the dynamic evolutionary processes in detail, molecular phylogenetic analysis of the host stinkbugs is also needed, which should be pursued in future studies.

### Molecular evolutionary rate and nucleotide composition among gut symbiotic bacteria of pentatomid stinkbugs

In the phylogeny, the gut symbiotic bacteria conserved in their host genera (ex. those of *Nezara* spp., *Menida* spp., *Homalogonia* spp., *Pentatoma* spp., *Scotinophara* spp., etc.) tended to exhibit elongated branches, whereas the gut symbiotic bacteria promiscuously intermingled with free-living bacteria tended to exhibit very short branches (Fig. 1). Relative rate tests of the 16S rRNA gene sequences confirmed these observations: molecular evolutionary rates were remarkably accelerated (K1/K2 values 1.5 to 2.4, *P* values < 10^−5^) in the gut symbiotic bacteria of *Menida* spp., *Homalogonia* spp., and *Pentatoma* spp., and other long-branched gut symbiont lineages associated with a variety of pentatomid stinkbugs including *Gonopsis affinis*, *Piezodorus hybneri*, *Niphe elongata*, *Erthesina fullo*, *Hermolaus amurensis*, *Halyomorpha halys*, and *Lelia decapunctata*; molecular evolutionary rates were moderately accelerated (K1/K2 values 1.1 to 1.6, *P* values 0.001–0.04) in the gut symbiotic bacteria of *Scotinophara* spp., *Nezara* spp., *Palomena angulosa*, *Rhynchocoris humeralis*, *Glaucias subpunctatus* and *Carpocoris purpureipennis*; and no accelerated molecular evolution was detected (K1/K2 values around 1, *P* values > 0.05) in the gut symbiotic bacteria of promiscuous type from *Chalazonotum ishiharai*, *Bathycoelia indica*, *Vitellus orientalis*, *Carbula abbreviata*, *Alcimocoris japonensis*, *Laprius gastricus*, *Aelia fieberi*, *Graphosoma rubrolineatum*, *Dybowskyia reticulata*, *Agonoscelis femoralis*, *Rubiconia intermedia*, and *Paraholcostethus breviceps* (Additional file 3; Fig. 1). Notably, AT contents of the 16S rRNA gene sequences exhibited overall correlation with the categories: 46.3 to 49.5% for the highly accelerated sequences; 44.3 to 45.7% for the moderately accelerated sequences; and 43.9 to 44.9% for the sequences exhibiting no acceleration (Additional file 1).

### Molecular evolutionary rate and nucleotide composition among gut symbiotic bacteria of other stinkbug groups and allied free-living γ-proteobacteria

Furthermore, 16S rRNA gene sequences of gut symbiotic bacteria of stinkbugs representing the Pentatomidae and other families published in previous studies, and also those of allied free-living γ-proteobacteria, all of which are members of the Enterobacteriaceae (Fig. 1), were similarly subjected to molecular evolutionary analyses (Additional file 4). Relative rate tests revealed the following patterns: in the genome-reduced and co-speciating gut symbiotic bacteria from the stinkbug families Plataspidae, Acanthosomatidae and Urostylididae [11, 12, 18], molecular evolutionary rates were extremely accelerated (K1/K2 values 3.4–5.0, *P* values < 10^−7^) with extremely high AT contents (>50.3%); in the genome-reduced gut symbiotic bacterium from the stinkbug family Parastrachiidae [37], a highly accelerated molecular evolutionary rate was observed (K1/K2 value 1.5, *P* value < 10^−4^) with extremely high AT content (51.0%); in the uncultivable gut symbiotic bacteria of pentatomid stinkbugs, some species (*Eurydema* spp. and *Halyomorpha halys*) [15, 28] exhibited remarkably high molecular evolutionary rates (K1/K2 values 1.8–2.0, *P* values < 10^−7^) with relatively high AT contents (48.1–48.6%), other species (*Nezara viridula* and *Plautia stali* type A) [14, 22] showed moderately high molecular evolutionary rates (K1/K2 values 1.1–1.6, *P* values around 0.02) with low AT contents (44.7–45.7%), and other species (*Plautia splendens* and *Plautia stali* B type) [21, 22] entailed no accelerated molecular evolution (K1/K2 values around 1.1, *P* values > 0.05) with low AT contents (43.9–44.3%); in the cultivable gut symbiotic bacteria of pentatomid stinkbugs (*Axiagastus rosmarus* C-E types and *Plautia stali* C-F types) [22], no accelerated molecular evolution was detected (K1/K2 values around 1.0, *P* values 0.3–1.0) with low AT contents (44.4–44.9%).

### Relationships between molecular evolutionary rate, nucleotide composition, genome size, and cultivability of gut symbiotic bacteria of pentatomid stinkbugs

**Fig. 2:**
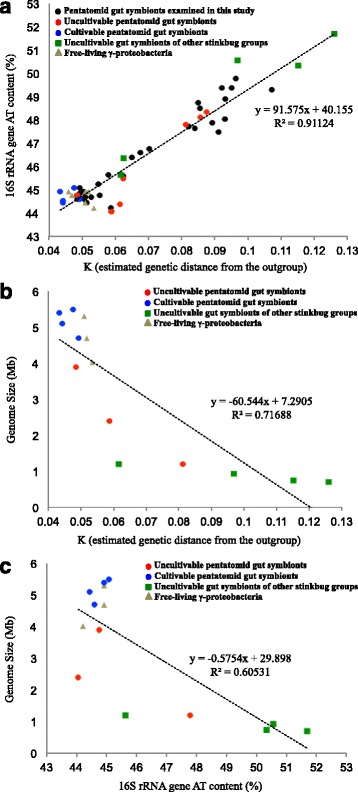
Relationships between molecular evolutionary rate, nucleotide composition, and genome size between gut symbiotic bacteria of pentatomid stinkbugs, gut symbiotic bacteria of other stinkbug groups, and allied free-living γ-proteobacteria. **a** K vs. AT content of 16S rRNA gene. **b** K vs. genome size. **c** AT content of 16S rRNA gene vs. genome size. K is defined as estimated genetic distance from the outgroup of “the stinkbug gut symbiont clade subjected to comparative evolutionary analyses” (see Fig. 1 and Additional file 5), reflecting the molecular evolutionary rate of the lineage

Previous studies have revealed that, through intimate and long-lasting host-symbiont co-evolution, obligate endocellular symbiotic bacteria of diverse insects tend to exhibit a characteristic genomic syndrome, including accelerated molecular evolution, AT-biased nucleotide composition, massive gene loss, and reduced genome size. This has been ascribed to stable and nutrition-rich endocellular environment and to strong population bottlenecks associated with the lifestyles of vertically-transmitted symbiotic bacteria [53–56]. Recently, remarkable genome reduction has also been identified among extracellular gut symbiotic bacteria of various stinkbugs [11, 12, 17, 18, 22, 28, 37, 41], elucidating that endocellularity cannot be the main driver of the symbiosis-associated reductive genome evolution [7, 11, 57]. In this study, a wide variety of gut symbiotic bacteria of pentatomid stinkbugs at different evolutionary stages, ranging from cultivable through uncultivable to genome-reduced, were found together with free-living bacteria and diverse gut symbiotic bacteria of other stinkbug families (Fig. 1), which provided an ideal opportunity to comparatively analyze the evolutionary processes underpinning the gut symbiotic associations. For that purpose, we focused on “the stinkbug gut symbiont clade subjected to comparative evolutionary analyses” (see Fig. 1 on the right side), defined an outgroup of the clade (*Yersinia pestis* KIM10+), and calculated the value K (genetic distance from the outgroup) for each member of the clade (Additional file 5), which enabled comparison of molecular evolutionary rates across all members of the clade. Figure 2a shows the relationship between K values and AT contents within the clade, indicating a fairly strong positive correlation (R = 0.955). On the plot, the gut symbionts of diverse pentatomid stinkbugs (black, red and blue) were scattered from the lower left (= low evolutionary rate and low AT content) to the upper right (= high evolutionary rate and high AT content). When uncultivable and genome-reduced gut symbionts of other stinkbug families (green) and free-living γ-proteobacteria (grey) were analyzed on the same plot, the former tended to be located on the upper right extreme (= highest evolutionary rate and highest AT content), whereas the latter were concentrated on the lower left extreme (= lowest evolutionary rate and lowest AT content). For some of the gut symbionts of pentatomid stinkbugs, we examined whether they are cultivable or not [14, 15, 21, 22, 28] (see Additional file 2). Interestingly, the uncultivable gut symbionts of pentatomid stinkbugs (red) tended to be distributed from the middle to the lower left, whereas the cultivable gut symbionts of pentatomid stinkbugs (blue) were concentrated on the lower left extreme as the free-living γ-proteobacteria. Figure 2b shows the relationship between K values and genome sizes within the clade, exhibiting a strong negative correlation (*R* = −0.847). In the plot, the uncultivable and genome-reduced gut symbionts of other stinkbug families (green) were located to the lower right extreme, the cultivable gut symbionts of pentatomid stinkbugs (blue) and the free-living γ-proteobacteria (grey) were concentrated to the upper left extreme, and the uncultivable gut symbionts of pentatomid stinkbugs (red) exhibited an intermediate distribution. Figure 2c shows the relationship between AT contents and K values within the clade, exhibiting a similar pattern to Fig. 2b with a strong negative correlation (*R* = −0.778).

### Multiple evolutionary origins and different evolutionary stages of gut symbiotic bacteria in the Pentatomidae

These results collectively highlight the following dynamic evolutionary aspects of the gut symbiotic bacteria in the stinkbug family Pentatomidae: (i) each of the pentatomid host species examined in this study is monosymbiotically associated with a specific γ-proteobacterium within the midgut symbiotic organ; (ii) gut symbiotic bacteria are of multiple evolutionary origins in the Pentatomidae, presumably originating from free-living γ-proteobacteria belonging to the Enterobacteriaceae repeatedly; (iii) some are of relatively old origin and stably maintained across closely related host species, some are of relatively recent origin and stably associated with a specific host species, and others are of promiscuous nature whose infections may be polymorphic within and/or between host species; (iv) stable gut symbiotic bacteria tend to exhibit uncultivability, accelerated molecular evolution, AT-biased nucleotide composition and reduced genome; (v) by contrast, promiscuous gut symbiotic bacteria tend to exhibit cultivability, non-accelerated molecular evolution, unbiased nucleotide composition and non-reduced genome, which are similar to free-living environmental bacteria; and (vi) a number of gut symbiont lineages at different evolutionary stages of different symbiotic intimacy coexist within the stinkbug family Pentatomidae.

## Conclusions

In conclusion, gut symbiotic bacteria have evolved repeatedly and dynamically within the stinkbug family Pentatomidae, which have plausibly entailed frequent acquisitions, losses, replacements, and transfers, and sometimes led to establishment of relatively stable host–symbiont associations. The remarkable diversity of host–symbiont relationships in the Pentatomidae provides an ideal arena for investigating the evolution of symbiosis not only experimentally but also theoretically. The mechanisms and processes underlying stability vs. promiscuity, vertical transmission vs. horizontal acquisition, cultivability vs. uncultivability, etc. among the gut symbiotic bacteria are of particular interest. For example, the stability of host–symbiont associations must be relevant to stable vertical transmission ensured by maternal deposition of symbiont-containing excretion onto the egg surface [21], and to selection of specific bacteria mediated by a symbiont sorting organ in the stinkbug midgut [58]. What ecological factors are relevant to the symbiont transmission mode (vertical, horizontal, or environmental) is an important theoretical issue [59]. It is unknown what biological functions underpin the beneficial roles of the diverse symbiotic bacteria for the host stinkbugs, to which genomic and physiological comparisons among the symbiotic bacteria may provide valuable insights [22, 57]. A number of pentatomid stinkbugs are notorious as agricultural pests [60], and biological understanding of their symbiotic bacteria may also contribute to their control and management. In this study, we describe the gut symbiotic bacteria of 35 Japanese pentatomid species, which account for over 40% of all pentatomid species reported from Japan [61]. In the world, some 5000 pentatomid stinkbugs have been described [62], and we suggest that a survey of their symbiotic microbial associates will lead to further discoveries of general relevance.

## Additional files

Additional file 1: Stinkbug samples used in this study. (XLSX 18 kb)
Additional file 2: Bacterial 16S rRNA gene sequences of gut symbionts of stinkbugs and free-living bacteria retrieved from the DNA databases and analyzed in this study. (XLSX 18 kb)
Additional file 3: Relative rate tests of 16S rRNA gene sequences of the gut symbionts of pentatomid stinkbugs in comparison with allied free-living bacteria. (DOCX 163 kb)
Additional file 4: Relative rate tests of 16S rRNA gene sequences of the cultivable and uncultivable stinkbug gut symbionts in comparison with allied free-living bacteria. (DOCX 152 kb)
Additional file 5: Bacterial taxa and their relevant parameters analyzed in Fig. 2. (DOCX 136 kb)
