# Supplementary material for: Recurrent evolution of gut symbiotic bacteria in pentatomid stinkbugs
Source: Zoological Lett. 2016 Nov 30;2:24. doi: 10.1186/s40851-016-0061-4 (PMC5131451; doi:10.1186/s40851-016-0061-4)
Supplement: Additional file 3: — Relative rate tests of 16S rRNA gene sequences of the gut symbionts of pentatomid stinkbugs in comparison with allied free-living bacteria. (DOCX 163 kb) [file 40851_2016_61_MOESM3_ESM.docx]

Additional file 3. Relative rate tests of 16S rRNA gene sequences of the gut symbionts of pentatomid stinkbugs in comparison with allied free-living bacteria.

| Lineage 1  (gut symbionts of stinkbugs)^a^ | Lineage 2  (allied free-living bacteria) | Outgroup | K1^b^ | K2^c^ | K1-K2 | K1/K2 | *P*-value^d^ |
| --- | --- | --- | --- | --- | --- | --- | --- |
| *Nezara antennata* | *Enterobacter cloacae* ATCC13047 | *Pantoea dispersa* LMG2603 | 0.039 | 0.024 | 0.014 | 1.585 | 0.020^*^ |
| *Palomena angulosa* | *Enterobacter cloacae* ATCC13047 | *Pantoea dispersa* LMG2603 | 0.037 | 0.024 | 0.013 | 1.515 | 0.041^*^ |
| *Menida violacea* | *Pantoea dispersa* LMG2603 | *Yersinia pestis* KIM10+ | 0.095 | 0.044 | 0.051 | 2.145 | 1.0 x 10^-7***^ |
| *Menida disjecta* | *Pantoea dispersa* LMG2603 | *Yersinia pestis* KIM10+ | 0.093 | 0.044 | 0.049 | 2.103 | 1.0 x 10^-7***^ |
| *Menida versicolor* | *Pantoea dispersa* LMG2603 | *Yersinia pestis* KIM10+ | 0.107 | 0.044 | 0.063 | 2.420 | 1.0 x 10^-7***^ |
| *Menida musiva* | *Pantoea dispersa* LMG2603 | *Yersinia pestis* KIM10+ | 0.085 | 0.044 | 0.041 | 1.918 | 1.0 x 10^-7***^ |
| *Homalogonia obtusa* | *Pantoea dispersa* LMG2603 | *Yersinia pestis* KIM10+ | 0.093 | 0.044 | 0.049 | 2.099 | 1.0 x 10^-7***^ |
| *Homalogonia confusa* | *Pantoea dispersa* LMG2603 | *Yersinia pestis* KIM10+ | 0.091 | 0.044 | 0.047 | 2.056 | 1.0 x 10^-7***^ |
| *Gonopsis affinis* | *Pantoea dispersa* LMG2603 | *Yersinia pestis* KIM10+ | 0.096 | 0.044 | 0.052 | 2.173 | 1.0 x 10^-7***^ |
| *Piezodorus hybneri* | *Pantoea dispersa* LMG2603 | *Yersinia pestis* KIM10+ | 0.085 | 0.044 | 0.041 | 1.931 | 1.0 x 10^-7***^ |
| *Niphe elongata* | *Pantoea dispersa* LMG2603 | *Yersinia pestis* KIM10+ | 0.092 | 0.044 | 0.048 | 2.080 | 1.0 x 10^-7***^ |
| *Erthesina fullo* | *Pantoea dispersa* LMG2603 | *Yersinia pestis* KIM10+ | 0.089 | 0.044 | 0.045 | 2.014 | 1.0 x 10^-7***^ |
| *Hermolaus amurensis* | *Pantoea dispersa* LMG2603 | *Yersinia pestis* KIM10+ | 0.084 | 0.044 | 0.040 | 1.894 | 1.0 x 10^-7***^ |
| *Halyomorpha halys* | *Pantoea dispersa* LMG2603 | *Yersinia pestis* KIM10+ | 0.082 | 0.044 | 0.038 | 1.855 | 1.0 x 10^-7***^ |
| *Pentatoma japonica* | *Pantoea dispersa* LMG2603 | *Yersinia pestis* KIM10+ | 0.068 | 0.044 | 0.023 | 1.527 | 2.4 x 10^-5***^ |
| *Pentatoma rufipes* | *Pantoea dispersa* LMG2603 | *Yersinia pestis* KIM10+ | 0.070 | 0.044 | 0.026 | 1.586 | 5.7 x 10^-6***^ |
| *Lelia decempunctata* | *Pantoea dispersa* LMG2603 | *Yersinia pestis* KIM10+ | 0.065 | 0.044 | 0.021 | 1.466 | 2.7 x 10^-5***^ |
| *Chalazonotum ishiharai* | *Pantoea dispersa* LMG2603 | *Yersinia pestis* KIM10+ | 0.049 | 0.044 | 0.004 | 1.096 | 0.159 |
| *Bathycoelia indica* | *Pantoea dispersa* LMG2603 | *Yersinia pestis* KIM10+ | 0.044 | 0.044 | 0 | 1 | 1 |
| *Rhynchocoris humeralis* | *Pantoea dispersa* LMG2603 | *Yersinia pestis* KIM10+ | 0.049 | 0.044 | 0.004 | 1.096 | 0.024^*^ |
| *Vitellus orientalis* | *Enterobacter ludwigii* K9 | *Yersinia pestis* KIM10+ | 0.049 | 0.046 | 0.003 | 1.075 | 0.351 |
| *Glaucias subpunctatus* | *Enterobacter ludwigii* K9 | *Yersinia pestis* KIM10+ | 0.055 | 0.046 | 0.009 | 1.195 | 0.016^*^ |
| *Carbula abbreviata* | *Enterobacter ludwigii* K9 | *Yersinia pestis* KIM10+ | 0.049 | 0.046 | 0.003 | 1.074 | 0.265 |
| *Alcimocoris japonensis* | *Enterobacter ludwigii* K9 | *Yersinia pestis* KIM10+ | 0.051 | 0.046 | 0.005 | 1.102 | 0.126 |
| *Laprius gastricus* | *Pantoea stewartii* ATCC8199 | *Yersinia pestis* KIM10+ | 0.049 | 0.051 | -0.002 | 0.967 | 0.648 |
| *Aelia fieberi* | *Pantoea ananatis* LMG20103 | *Yersinia pestis* KIM10+ | 0.059 | 0.052 | 0.007 | 1.133 | 0.077 |
| *Graphosoma rubrolineatum* | *Pantoea ananatis* LMG20103 | *Yersinia pestis* KIM10+ | 0.051 | 0.052 | -0.001 | 0.976 | 0.640 |
| *Dybowskyia reticulata* | *Pantoea ananatis* LMG20103 | *Yersinia pestis* KIM10+ | 0.051 | 0.052 | 0.000 | 0.992 | 0.879 |
| *Agonoscelis femoralis* | *Pantoea ananatis* LMG20103 | *Yersinia pestis* KIM10+ | 0.059 | 0.052 | 0.007 | 1.133 | 0.077 |
| *Carpocoris purpureipennis* | *Pantoea ananatis* LMG20103 | *Yersinia pestis* KIM10+ | 0.055 | 0.052 | 0.007 | 1.068 | 0.034^*^ |
| *Rubiconia intermedia* | *Pantoea ananatis* LMG20103 | *Yersinia pestis* KIM10+ | 0.051 | 0.052 | -0.001 | 0.983 | 0.844 |
| *Paraholcostethus breviceps* | *Pantoea ananatis* LMG20103 | *Yersinia pestis* KIM10+ | 0.053 | 0.052 | 0.001 | 1.017 | 0.849 |
| *Scotinophara horvathi* | *Yersinia pestis* KIM10+ | *Vibrio cholerae* MJ1236 | 0.107 | 0.087 | 0.020 | 1.224 | 0.007^**^ |
| *Scotinophara scottii* | *Yersinia pestis* KIM10+ | *Vibrio cholerae* MJ1236 | 0.113 | 0.087 | 0.025 | 1.290 | 0.00097^***^ |
| *Scotinophara lurida* | *Yersinia pestis* KIM10+ | *Vibrio cholerae* MJ1236 | 0.113 | 0.087 | 0.025 | 1.290 | 0.001^**^ |

^a^Scientific names of host stinkbugs are shown.

^b^Estimated mean distance between symbiont lineage and the last common ancestor of symbiont lineage and allied free-living bacterial lineage.

^c^Estimated mean distance between allied free-living bacterial lineage and the last common ancestor of symbiont lineage and allied free-living bacterial lineage.

^d^*P* values were estimated by the program RRTree [36] (^*^, *P* < 0.05; ^**^, *P* < 0.01; ^***^, *P* < 0.001). The analysis was performed using 1,260 nucleotide sites of the 16S rRNA gene sequences. In figure 1, significance levels of the *P*-values are indicated by asterisks beside the symbiont sequences from pentatomid species determined in this study in red.
