# Supplementary material for: Recurrent evolution of gut symbiotic bacteria in pentatomid stinkbugs
Source: Zoological Lett. 2016 Nov 30;2:24. doi: 10.1186/s40851-016-0061-4 (PMC5131451; doi:10.1186/s40851-016-0061-4)
Supplement: Additional file 4: — Relative rate tests of 16S rRNA gene sequences of the cultivable and uncultivable stinkbug gut symbionts in comparison with allied free-living bacteria. (DOCX 152 kb) [file 40851_2016_61_MOESM4_ESM.docx]

Additional file 4. Relative rate tests of 16S rRNA gene sequences of the cultivable and uncultivable stinkbug gut symbionts in comparison with allied free-living bacteria.

| Lineage 1^a^  (gut symbionts of stinkbugs) | Lineage 2  (allied free-living bacteria) | Outgroup | K1^b^ | K2^c^ | K1-K2 | K1/K2 | *P*-value^d^ |
| --- | --- | --- | --- | --- | --- | --- | --- |
| Uncultivable gut symbionts of pentatomid stinkbugs | | |  |  |  |  |  |
| *Eurydema dominulus* | *Pantoea dispersa* LMG2603 | *Yersinia pestis* KIM10+ | 0.086 | 0.044 | 0.041 | 1.934 | 1.0 x 10^-7***^ |
| *Eurydema rugosa* | *Pantoea dispersa* LMG2603 | *Yersinia pestis* KIM10+ | 0.088 | 0.044 | 0.043 | 1.976 | 1.0 x 10^-7***^ |
| *Halyomorpha halys*^e^ | *Pantoea dispersa* LMG2603 | *Yersinia pestis* KIM10+ | 0.081 | 0.044 | 0.037 | 1.834 | 3.5 x 10^-7***^ |
| *Nezara viridula* | *Enterobacter cloacae* ATCC13047 | *Pantoea dispersa* LMG2603 | 0.039 | 0.024 | 0.014 | 1.585 | 0.023^*^ |
| *Plautia splendens* | *Erwinia pyrifoliae* DSM12163 | *Yersinia pestis* KIM10+ | 0.061 | 0.054 | 0.008 | 1.146 | 0.083 |
| *Plautia stali*, A type | *Pantoea dispersa* LMG2603 | *Yersinia pestis* KIM10+ | 0.049 | 0.044 | 0.004 | 1.096 | 0.024^*^ |
| *Plautia stali,* B type | *Erwinia pyrifoliae* DSM12163 | *Yersinia pestis* KIM10+ | 0.059 | 0.054 | 0.005 | 1.097 | 0.278 |
| Cultivable gut symbionts of pentatomid stinkbugs | | |  |  |  |  |  |
| *Axiagatus rosmatus*, C type | *Pantoea dispersa* LMG2603 | *Yersinia pestis* KIM10+ | 0.044 | 0.044 | 0 | 1 | 1 |
| *Axiagatus rosmatus*, D type | *Enterobacter ludwigii* K9 | *Yersinia pestis* KIM10+ | 0.048 | 0.046 | 0.002 | 1.037 | 0.527 |
| *Axiagatus rosmatus*, E type | *Enterobacter ludwigii* K9 | *Yersinia pestis* KIM10+ | 0.043 | 0.046 | -0.003 | 0.945 | 0.400 |
| *Plautia stali*, C type | *Pantoea dispersa* LMG2603 | *Yersinia pestis* KIM10+ | 0.044 | 0.044 | 0 | 1 | 1 |
| *Plautia stali*, D type | *Enterobacter ludwigii* K9 | *Yersinia pestis* KIM10+ | 0.048 | 0.046 | 0.002 | 1.037 | 0.527 |
| *Plautia stali*, E type | *Enterobacter ludwigii* K9 | *Yersinia pestis* KIM10+ | 0.043 | 0.046 | -0.003 | 0.945 | 0.400 |
| *Plautia stali*, F type | *Pantoea ananatis* LMG20103 | *Yersinia pestis* KIM10+ | 0.049 | 0.052 | -0.003 | 0.951 | 0.337 |
| Uncultivable gut symbionts of other stinkbug groups | | |  |  |  |  |  |
| Cydnidae |  |  |  |  |  |  |  |
| *Adomerus rotundus* | *Pantoea agglomerans* DSM3493 | *Yersinia pestis* KIM10+ | 0.062 | 0.047 | 0.015 | 1.313 | 0.004^**^ |
| *Adomerus triguttulus* | *Pantoea agglomerans* DSM3493 | *Yersinia pestis* KIM10+ | 0.062 | 0.047 | 0.016 | 1.332 | 0.005^**^ |
| Parastrachiidae |  |  |  |  |  |  |  |
| *Parastrachia japonensis*^f^ | *Yersinia pestis* KIM10+ | *Vibrio cholera* MJ1236 | 0.130 | 0.087 | 0.042 | 1.485 | 3.8 x 10^-5***^ |
| Acanthosomatidae |  |  |  |  |  |  |  |
| *Elasmostethus humeralis*^g^ | *Enterobacter cloacae* ATCC13047 | *Pantoea dispersa* LMG2603 | 0.088 | 0.026 | 0.063 | 3.436 | 1.0 x 10^-7***^ |
| Plataspidae |  |  |  |  |  |  |  |
| *Megacopta punctatissima*^h^ | *Enterobacter cloacae* ATCC13047 | *Pantoea dispersa* LMG2603 | 0.101 | 0.024 | 0.076 | 4.142 | 1.0 x 10^-7***^ |
| Urostylididae |  |  |  |  |  |  |  |
| *Urostylis westwoodii*^i^ | *Enterobacter cloacae* ATCC13047 | *Pantoea dispersa* LMG2603 | 0.122 | 0.024 | 0.098 | 5.015 | 1.0 x 10^-7***^ |

^a^Scientific names of host stinkbugs are shown.

^b^Estimated mean distance between symbiont lineage and the last common ancestor of symbiont lineage and allied free-living bacterial lineage.

^c^Estimated mean distance between allied free-living bacterial lineage and the last common ancestor of symbiont lineage and allied free-living bacterial lineage.

^d^*P* values were estimated by the program RRTree [36] (^*^, *P* < 0.05; ^**^, *P* < 0.01; ^***^, *P* < 0.001). The analysis was performed using 1,260 nucleotide sites of the 16S rRNA gene sequences, except for the gut symbiont of *Elasmostehus humeralis* for which only 1,191 nucleotide sites were available. In figure 1, significance levels of the *P*-values are indicated by asterisks beside the symbiont sequences of pentatomid stinkbugs determined in previous studies in green, and beside the symbiont sequences from other stinkbugs determined in previous studies in blue.

^e^*Candidatus* Pantoea carbekii [28].

^f^*Candidatus* Benitsuchiphilus tojoi [37].

^g^*Candidatus* Rosenkranzia clausaccus [12].

^h^*Candidatus* Ishikawaella capsulata [11].

^i^*Candidatus* Tachikawaea gelatinosa [18].
