# Supplementary material for: Recurrent evolution of gut symbiotic bacteria in pentatomid stinkbugs
Source: Zoological Lett. 2016 Nov 30;2:24. doi: 10.1186/s40851-016-0061-4 (PMC5131451; doi:10.1186/s40851-016-0061-4)
Supplement: Additional file 5: — Bacterial taxa and their relevant parameters analyzed in Fig. 2. (DOCX 136 kb) [file 40851_2016_61_MOESM5_ESM.docx]

Additional file 5. Bacterial taxa and their relevant parameters analyzed in Figure 2.

| Taxon | K^a^ | AT  (%) | Cultiva-bility | Genome size (Mb) | Accession number |
| --- | --- | --- | --- | --- | --- |
| Aligned 16S rRNA gene sequences of gut symbiotic bacteria of pentatomid stinkbugs used in the phylogenetic analyses and relative rate tests | | | | | |
| *Nezara antennata* | 0.062 | 45.58 | – | – | LC168530 |
| *Palomena angulosa* | 0.058 | 45.63 | – | – | LC168536 |
| *Menida violacea* | 0.095 | 49.37 | – | – | LC168526 |
| *Menida disjecta* | 0.093 | 48.89 | – | – | LC168520 |
| *Menida versicolor* | 0.107 | 49.29 | – | – | LC168525 |
| *Menida musiva* | 0.085 | 48.73 | – | – | LC168523 |
| *Homalogonia obtusa* | 0.093 | 48.02 | – | – | LC168516 |
| *Homalogonia confusa* | 0.091 | 47.76 | – | – | LC168514 |
| *Gonopsis affinis* | 0.096 | 49.76 | – | – | LC168598 |
| *Piezodorus hybneri* | 0.085 | 48.49 | – | – | LC168544 |
| *Niphe elongata* | 0.092 | 49.37 | – | – | LC168535 |
| *Erthesina fullo* | 0.089 | 47.86 | – | – | LC168492 |
| *Hermolaus amurensis* | 0.084 | 47.62 | – | – | LC168511 |
| *Pentatoma japonica* | 0.068 | 46.59 | – | – | LC168542 |
| *Pentatoma rufipes* | 0.070 | 46.75 | – | – | LC168543 |
| *Lelia decempunctata* | 0.065 | 46.39 | – | – | LC168518 |
| *Chalazonotum ishiharai* | 0.049 | 44.76 | – | – | LC168491 |
| *Bathycoelia indica* | 0.044 | 44.52 | – | – | LC168483 |
| *Rhynchocoris humeralis* | 0.049 | 44.6 | – | – | LC168557 |
| *Vitellus orientalis* | 0.049 | 45.08 | – | – | LC168565 |
| *Glaucias subpunctatus* | 0.055 | 45.24 | – | – | LC168580 |
| *Carbula abbreviata* | 0.049 | 44.84 | – | – | LC168485 |
| *Alcimocoris japonensis* | 0.051 | 44.92 | – | – | LC168568 |
| *Laprius gastricus* | 0.049 | 44.6 | – | – | LC168517 |
| *Aelia fieberi* | 0.059 | 44.21 | – | – | LC168475 |
| *Graphosoma rubrolineatum* | 0.051 | 44.6 | – | – | LC168602 |
| *Dybowskyia reticulata* | 0.051 | 44.44 | – | – | LC168578 |
| *Agonoscelis femoralis* | 0.059 | 44.05 | – | – | LC168481 |
| *Carpocoris purpureipennis* | 0.055 | 44.76 | – | – | LC168490 |
| *Rubiconia intermedia* | 0.051 | 44.68 | – | – | LC168558 |
| *Paraholcostethus breviceps* | 0.053 | 44.68 | – | – | LC168513 |
| Aligned 16S rRNA gene sequences of gut symbiotic bacteria of pentatomid stinkbugs retrieved from the DNA databases | | | | | |
| *Axiagatus rosmatus*, C type | 0.044 | 44.52 | Yes | – | LC007846 |
| *Axiagatus rosmatus*, D type | 0.048 | 45.08 | Yes | – | LC007845 |
| *Axiagatus rosmatus*, E type | 0.043 | 44.92 | Yes | – | LC007847 |
| *Eurydema dominulus* | 0.086 | 48.10 | No | – | AB650524 |
| *Eurydema rugosa* | 0.088 | 48.33 | No | – | AB650515 |
| *Halyomorpha halys*^b^ | 0.081 | 47.78 | No | 1.2 | CP010907 |
| *Nezara viridula* | 0.062 | 45.58 | No | – | AB636641 |
| *Plautia splendens* | 0.061 | 44.37 | No | – | LC012471 |
| *Plautia stali*, A type | 0.049 | 44.76 | No | 3.9 | LC007607 |
| *Plautia stali,* B type | 0.059 | 44.05 | No | 2.4 | LC007758 |
| *Plautia stali*, C type | 0.044 | 44.44 | Yes | 5.1 | LC007809 |
| *Plautia stali*, D type | 0.048 | 45.08 | Yes | 5.5 | LC007781 |
| *Plautia stali*, E type | 0.043 | 44.92 | Yes | 5.4 | LC007826 |
| *Plautia stali*, F type | 0.049 | 44.60 | Yes | 4.7 | LC007726 |
| Aligned 16S rRNA gene sequences of uncultivable gut symbiotic bacteria of other stinkbug groups retrieved from the DNA databases | | | | | |
| *Adomerus rotundus* | 0.062 | 45.63 | No | 1.2 | AB703086 |
| *Adomerus triguttulus* | 0.062 | 46.35 | No | – | AB703083 |
| *Elasmostethus humeralis*^c^ | 0.097 | 50.55 | No | 0.93 | AB368823 |
| *Megacopta punctatissima*^d^ | 0.115 | 50.32 | No | 0.75 | AP010872 |
| *Urostylis westwoodii*^e^ | 0.126 | 51.67 | No | 0.71 | AP014521 |
| Aligned 16S rRNA gene sequences of allied free-living γ-proteobacteria retrieved from the DNA databases | | | | | |
| *Enterobacter ludwigii* K9 | 0.046 | 44.92 | Yes | – | EF175735 |
| *Erwinia pyrifoliae* DSM12163 | 0.054 | 44.21 | Yes | 4.03 | NC_017390 |
| *Enterobacter cloacae* ATCC13047 | 0.051 | 44.92 | Yes | 5.31 | CP001918 |
| *Pantoea agglomerans* DSM3493 | 0.047 | 44.75 | Yes | – | NR_041978 |
| *Pantoea ananatis* LMG20103 | 0.052 | 44.92 | Yes | 4.70 | NC_013956 |
| *Pantoea dispersa* LMG2603 | 0.044 | 44.47 | Yes | – | DQ504305 |
| *Pantoea stewartii* ATCC8199 | 0.051 | 44.44 | Yes | – | FJ611852 |

^a^Estimated genetic distance from the outgroup (*Yersinia pestis* KIM10+) in terms of number of substitutions per site based on Kimura’s 2-parameter distance.

^b^*Candidatus* Pantoea carbekii [28].

^c^*Candidatus* Rosenkranzia clausaccus [12].

^d^*Candidatus* Ishikawaella capsulata [11].

^e^*Candidatus* Tachikawaea gelatinosa [18].
